# Longitudinal realist evaluation of the Dementia PersonAlised Care Team (D-PACT) intervention: protocol

**DOI:** 10.3399/BJGPO.2023.0019

**Published:** 2023-07-12

**Authors:** Hannah Wheat, Lauren Weston, Tomasina M Oh, Sarah Morgan-Trimmer, Wendy Ingram, Sarah Griffiths, Rod Sheaff, Paul Clarkson, Antonieta Medina-Lara, Crispin Musicha, Stuart Spicer, Obioha Ukoumunne, Victoria Allgar, Siobhan Creanor, Michael Clark, Cath Quinn, Alex Gude, Rose McCabe, Saqba Batool, Lorna Smith, Debra Richards, Hannah Shafi, Bethany Warwick, Reena Lasrado, Basharat Hussain, Hannah Jones, Sonia Dalkin, Angela Bate, Ian Sherriff, Louise Robinson, Richard Byng

**Affiliations:** 1 Community and Primary Care Research Group, University of Plymouth, Plymouth, UK; 2 Department of Health and Community Sciences, University of Exeter, Exeter, UK; 3 Peninsula Clinical Trials Unit, University of Plymouth, Plymouth, UK; 4 Centre for Ageing Population Studies, University College London, London, UK; 5 Peninsula Medical School, Plymouth, UK; 6 Social Care and Society, University of Manchester, Manchester, UK; 7 Health Economics Group, University of Exeter, Exeter, UK; 8 Medical Statistics, University of Plymouth, Plymouth, UK; 9 NIHR ARC South West Peninsula, Department of Health and Community Sciences, Faculty of Health and Life Sciences, University of Exeter, Exeter, UK; 10 Exeter Clinical Trials Unit, University of Exeter, Exeter, UK; 11 Care Policy and Evaluation Centre, London School of Economics and Political Science, London, UK; 12 School of Health and Psychological Sciences, University of London, London, UK; 13 Faculty of Health and Life Science, Northumbria University, London, UK; 14 Population Health Sciences Institute, Newcastle University, Newcastle upon Tyne, UK

**Keywords:** primary health care, dementia, personalised care, caregivers, realist evaluation

## Abstract

**Background:**

Different dementia support roles exist but evidence is lacking on which aspects are best, for whom, and in what circumstances, and on their associated costs and benefits. Phase 1 of the Dementia PersonAlised Care Team programme (D-PACT) developed a post-diagnostic primary care-based intervention for people with dementia and their carers and assessed the feasibility of a trial.

**Aim:**

Phase 2 of the programme aims to 1) refine the programme theory on how, when, and for whom the intervention works; and 2) evaluate its value and impact.

**Design & setting:**

A realist longitudinal mixed-methods evaluation will be conducted in urban, rural, and coastal areas across South West and North West England where low-income or ethnic minority populations (for example, South Asian) are represented. Design was informed by patient, public, and professional stakeholder input and phase 1 findings.

**Method:**

High-volume qualitative and quantitative data will be collected longitudinally from people with dementia, carers, and practitioners. Analyses will comprise the following: 1) realist longitudinal case studies; 2) conversation analysis of recorded interactions; 3) statistical analyses of outcome and experience questionnaires; 4a) health economic analysis examining costs of delivery; and 4b) realist economic analysis of high-cost events and ‘near misses’. All findings will be synthesised using a joint display table, evidence appraisal tool, triangulation, and stakeholder co-analysis.

**Conclusion:**

The realist evaluation will describe how, why, and for whom the intervention does or does not lead to change over time. It will also demonstrate how a non-randomised design can be more appropriate for complex interventions with similar questions or populations.

## How this fits in

UK policy recognises the need for improved dementia care, post-diagnosis. Dementia support workers (DSWs) are valued by people with dementia and carers, but their role and availability vary substantially. Decision makers need evidence to support decisions on what type of dementia support to provide and to inform their workforce strategies. This evidence not only needs to detail what outcomes it achieves, but also how it works, for whom, and in what circumstances, as this will not only support commissioning decisions but also effective implementation, enabling modification, where appropriate, to ensure underserved communities can also benefit. A primary care model of dementia support was developed for evaluation owing to the desire and recognised need for coordinated, ongoing, and collaborative care.

## Introduction

The term ‘dementia’ describes a progressive set of symptoms that includes loss of short-term memory and problem-solving ability, communication problems, and loss of visuospatial skills. More than 850 000 people in the UK live with dementia and this number is predicted to increase to around 1.1 million by 2025. With an ageing population and 72% of individuals who have dementia also living with another medical condition or disability, a significant impact can be expected on the NHS and social care services.^1^ The need to *'deliver integrated and effective services that meet the needs of people with dementia and their families and carers'* was set out by the UK Department for Health and Social Care, along with an ambition to ensure that appropriate evidence is available across health and social care on best practice in post-diagnostic care.^2^ However, little is known about what kind of support is likely to be feasible, acceptable, and have a positive impact, especially for historically underserved communities who may face even more barriers accessing services.^3^

Feedback from people with dementia and their informal carers suggests that current access to post-diagnostic dementia support and the model of support provided is extremely variable. They report often finding access to support services stressful and challenging, and describe the *'maze-like'* services landscape, the limited and variable availability and remits of services, and the struggle to make headway.^4^ People with dementia and informal carers have identified that they strongly desire access to a single person, to aid the coordination of care throughout the dementia trajectory.^5,6^

There are a variety of dementia support roles within the NHS and social care services (for example, DSWs, dementia navigators, and dementia advisers) in different settings around the UK. While patients value these roles,^7,8^ there is lack of evidence as to the most effective aspects of support, who is most likely to benefit, where it would be best delivered, and what the costs and the health benefits of these support roles might be.^2–5^ Such information is needed by decision makers (commissioners and providers) when considering whether to invest in these dementia support services. There is, therefore, a need to understand, in relation to the DSW role, *'what works, for whom, in what circumstances and why'*.^9^

The 5-year D-PACT programme is funded by the National Institute for Health and Care Research to address these knowledge gaps. It has developed, and is about to evaluate, an intervention for people with dementia and carers that provides ongoing post-diagnostic support in the form of a DSW, embedded within primary care.

### The D-PACT intervention

There are four core steps within the D-PACT person-centred coaching model, delivered by DSWs, embedded within primary care, to people with dementia and carers (if they also wish to receive support):

1. support the people you are working with to identify what is important to them;
2. agree what will be discussed during current meetings (and if necessary what will be discussed in later meetings);
3. explore possible actions; and
4. create a plan (in terms of immediate actions but also in terms of the future, for example, advanced care planning statements and contingency plans). These steps do not have to be enacted in a linear way and each step will be returned to over time.

What the DSWs provide support on is co-agreed with participants based on their individual needs, but key areas the DSWs are likely to provide support for include: physical health and wellbeing; advanced care planning; social engagement and relationships; identifying opportunities for financial support/care aid; support during times of transition (for example, entry/discharge into/from hospital, new care team involved in care, respite, and permanent placements within care/nursing homes); connecting with community resources; and maintaining activities the individual enjoys. The length/intensity of the intervention is flexible, depending on peoples’ individual needs and preferences. The person with dementia and carer can determine how much they meet with the DSW over the 12-month period and how they interact with them — there is no lower or upper limit. The 12-month cap to DSW support was imposed in the project to allow a suitable time for analysis, but if the intervention was delivered outside of a research context, the DSW support would be ongoing until the person they supported no longer wanted, or were unable to receive, the support.

### Intervention development

The D-PACT intervention development was designed to be conducted over two phases, informed by the framework for realist evaluation proposed by Pawson and Tilley.^9^

Stage 1 is the development phase and consits of a) development of the initial programme theory (IPT) underlying the D-PACT intervention; and b) refinement of an elaborated programme theory (EPT) through piloting the intervention-in-development. Stage 2 is the evaluation, which will be rigorous testing of the programme theory, with more participants in various settings, so resulting evidence can be used to corroborate, refute, or extend the understanding of the EPT during analysis.

Completion of the two phases will lead to a finalised version of the theory. The term ‘finalised’ refers to it being the last version of the programme theory that the project produces. It does not suggest that the theory cannot be enhanced further, through future studies. At the time of writing (January 2023), the first phase has been completed, and the second phase is in progress (due to be completed in February 2024).

#### A realist approach to evaluation

Realist evaluation is a form of theory-driven evaluation used to understand if, how, for whom, and under what circumstances an intervention ‘works’ to produce intended outcomes.^9^ It is increasingly used for complex care interventions owing to the focus on understanding how interventions work for different people and why outcomes may or may not be attained in different contexts. Realist evaluations seek to uncover how intervention outcomes (O) are produced by examining the mechanisms (M) intended to produce them and the various contexts (C) that interact to enable or constrain the mechanism taking effect.^9,10^ The ways in which particular contexts and mechanisms produce outcomes are conceptualised in explanatory programme theories, commonly articulated through ‘context-mechanism-outcome’ (CMO) configurations. The CMO heuristic is often used as a framework in realist evaluation, transforming ‘implicit’ causal mechanisms into explicit programme theory statements. This guides what and how data are collected, the analytic process, and interpretation of evidence through the realist evaluation process. The programme theory (consisting of all the CMO statements) is developed iteratively, cycling between 1) theory development (that is, generating a working theory or hypothesis); 2) theory verification (that is, hypothesis or theory testing throughout data collection); and 3) theory refinement (that is, refining the hypothesis or theory based on emerging data).

### Evaluation aims

There are two core aims of the evaluation:

1. To test and refine the D-PACT programme theory to better understand:
  - how various components (for example, supporting disclosure; enhancing empowerment; and developing shared understanding and facilitating collaboration with other professionals) of the delivered intervention work, in what context they work (for example, stage of illness, and organisational, cultural, or geographical), for whom (that is, people with dementia and their informal carers with diverse personal, socioeconomic status, and cultural understanding circumstances), and what outcomes they generate (proximal and distal) for people with dementia and carers; and
  - how the facilitative actions supporting the delivery of the intervention (including training, supervision, and peer support) work, when it works (for example, in what organisational context), for whom (that is, DSWs from diverse professional backgrounds with different learning preferences and so on), and what outcomes they generate (proximal and distal) for DSWs.

**Figure 1.**
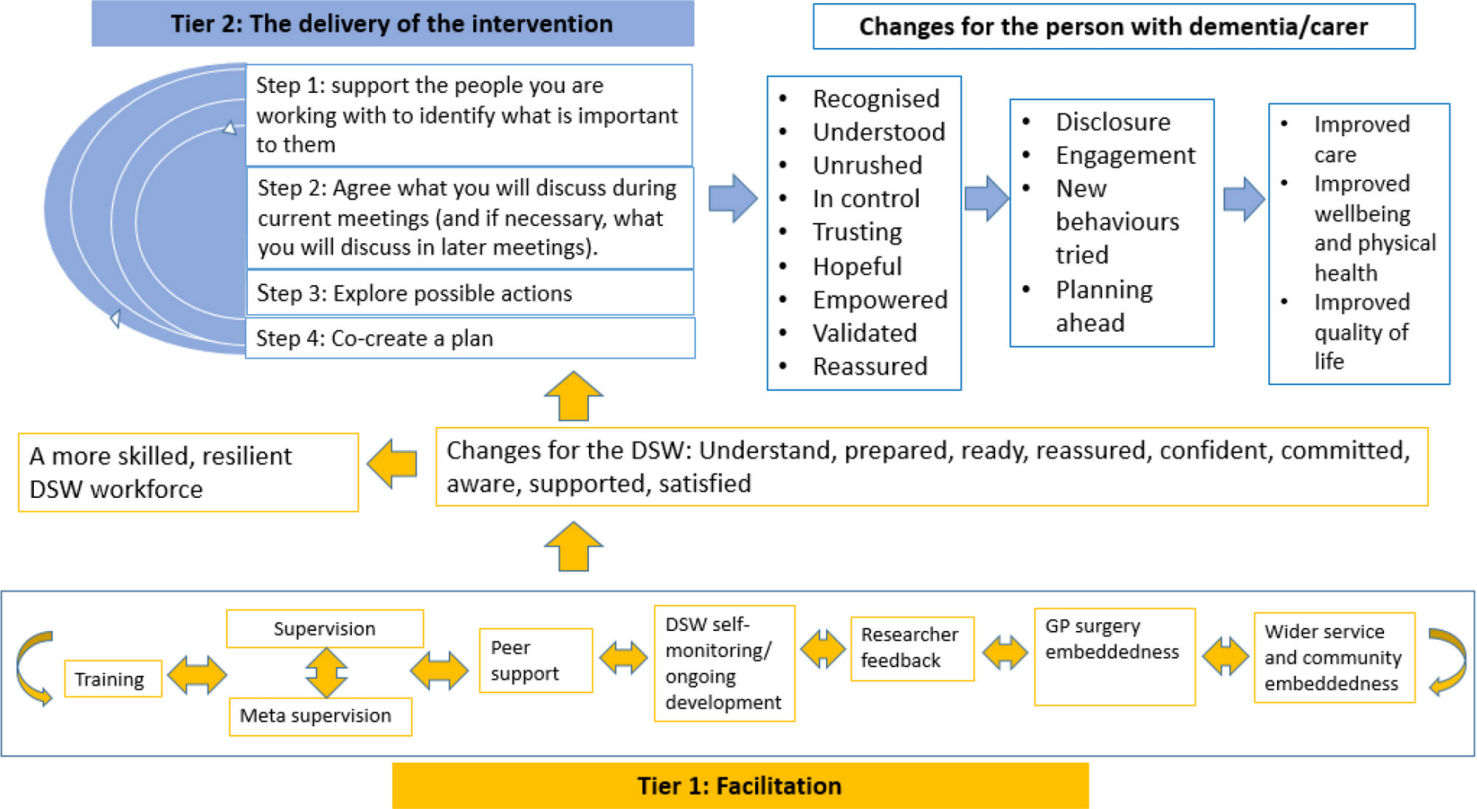
A visualisation of the D-PACT programme theory. D-PACT = Dementia PersonAlised Care Team. DSW = dementia support worker

Figure 1 visualises how the two tiers of the programme theory intertwine to make the intervention work.

2. To examine the potential value and impact of the intervention.

Here, the NHS Impact Framework’s definition of value and impact is adopted, where 'value' refers to the importance, worth, or usefulness of the intervention, and 'impact' to the intervention having an effect, influence, or resulting in changes, whether positive or negative.^11^

## Method

### Study design

Consistent with realist principles, a mixed-method approach to longitudinal data collection will enable the EPT to be tested and refined, and evidence both the proximal outcomes and distal outcomes of the intervention. The conduct and reporting of the evaluation will be guided by the Realist And Meta-narrative Evidence Synthesis: Evolving Standards (RAMESES II) reporting standards^12^ and the Medical Research Council (MRC).^13,14^

Members of the project’s peer research group and professional stakeholders have informed^15^ (and will continue to inform) the development of the research materials and processes.

Supplementary Box S1 details the data sources for the evaluation. Data and trials managers at the Peninsula Clinical Trials Unit will have oversight of data captured at screening, recruitment, and baseline, and in the case report files at follow-up. Central monitoring of data will be performed to include assessments of participant recruitment rates, attrition rates, data completeness, data quality, and protocol non-compliance. Qualitative data will be stored and managed on a secure file-saving platform, hosted at the University of Plymouth, which only research team members will have access to. Following completion of study data analysis, the sponsor will be responsible for archiving the study data and essential documents in a secure location for at least 5 years after the end of the analysis.

### Setting

The aim is to recruit 18 GP practices across two geographical regions, the South West (SW) — specifically Devon — and North West (NW) — specifically Greater Manchester — of England. Selection criteria for these practices include the following:

- interest in the project;
- localisation (urban, rural, and coastal areas across SW and NW England with higher representation of ethnic minority populations, particularly South Asian or communities with high deprivation); and
- a reasonable number of patients on the practice’s Quality and Outcomes Framework dementia register.

### Participants

People with dementia, their informal carers (if available and willing), and practitioners will be recruited. The latter group will comprise DSWs (minimum of 5), their supervisors (minimum of 4), and primary care staff members (minimum of 15) from participating GP surgeries will have the opportunity to participate in interviews, observations, and feedback sessions.

It is planned to recruit up to 180 people with dementia and up to 160 informal carers. Patients who have a diagnosis of dementia and registered at one of the participating practices will be eligible for study inclusion. People with dementia without carers are eligible for this study, as are those who lack capacity to consent (an under-represented group in dementia studies). Patients will not be eligible for study inclusion if they are:

- receiving substantial support from Community and Mental Health Teams (CMHTs);
- have open safeguarding referrals and ongoiong planned CMHT care;
- have a long-standing history of mental health difficulties and currently receiving care from other mental health teams; and/or
- are diagnosed with an end-stage physical health problem (for example, cancer and severe heart failure) with substantive multidisciplanry palliative and/or end-of-life care in place.

Final decision on study eligibility will ultimately lie with clinically qualified staff.

A minimum (90 people with dementia) to maximum (*n* = 180) range was chosen for pragmatic reasons, including uncertainty about recruitment rates. In brief, the minimum sample size allows the study to have enough variety in terms of patient cohorts, and fulfil requirements around the DSW’s caseload (45–55 patients). This figure was chosen based on the following three key criteria: 1) current DSW caseloads;^16^ 2) the objective of testing delivery of D-PACT with people with a wide range of characteristics (socioeconomic background, ethnic group, sex, dementia stage, and frailty) the time it would take for DSWs to provide data to the evaluation; and 3) to test the intervention theory around teamworking (at least two DSWs) and peer support with various types of primary care networks. The minimum target takes account of likely participant attrition in a longitudinal study (involving people with dementia and, in some cases, older carers), which aims to track changes over time and provides the study with a more accurate measure of ‘reach’; for example, the level of engagement of various groups that often miss out on care, and not just overall total.

The maximum recruitment will allow the study to test the ability of DSWs to provide care at a higher caseload and would further enhance heterogeneity of participants in order to better test intervention theory. While the sample size was not calculated statistically, a sample of at least 90–180 participants was estimated to provide a robust assessment of baseline and follow-up scores on measures.

Participants will be recruited either by 1) a proactive person-centred approach by embedded researchers, designed to reach as many people with dementia as possible (four-stage approach, described elsewhere [in a forthcoming manuscript])^17^ and reduce the burden of recruitment on primary care; or 2) responsive recruitment, where potentially eligible people with dementia can enquire about the study directly if they have seen advertisements, heard about it, or been approached or referred by their primary, secondary, and adult care teams, or community advocates.

### Analysis

The following four strands of analysis will be applied to the data: 1) longitudinal individual-level case studies; 2) conversation analysis on recorded interactions; 3) statistical analysis of outcome and experience measures; 4a) health economic analysis of the cost of delivery; and 4b) realist economic analysis of low-frequency, high-cost events and 'near misses'. These strands will then be synthesised in a joint display table;^18^ analyses will apply an evidence appraisal tool, triangulation, and stakeholder co-analysis. The project peer research group members will also be asked to attend co-analysis sessions and to co-design the dissemination plan and certain outputs.

#### 1. Longitudinal individual-level case studies

##### Units of analysis

For tier 1, facilitation tier of the programme theory: to refine understanding on how, when, and for whom individual components of support trigger mechanisms and outcomes for DSWs (the facilitation tier of the programme theory) the evaluation will conduct individual cases studies for each DSW (minimum of 5). Please see Box 1 for an example of a programme theory statement from the facilitation tier.

Box 1 **Example of a CCMO statement from the facilitation tier (under the ‘training’ domain). CCMO = context – (intervention) component – mechanism – outcome. DSW = dementia support worker.**

**Context:** IF a DSW has some experience delivering care for individuals with dementia but has gaps in training/experience on some of the core components of the intervention)

**Component:** IF the trainer delivers initial and top-up training through multiple formats, which facilitates synchronous and asynchronous learning

**Mechanisms:** THEN the DSW will feel supported to learn in a way that works for them

**Proximal Outcomes:** THEN the DSW will engage more in the training package

**Distal Outcomes:** THEN the DSW will become a more skilled practitioner within the field of personalised dementia care

For tier 2, delivery tier of the programme theory: to refine understanding of how the delivery of individual intervention components of the intervention, within different contexts, trigger mechanisms and (both proximal and distal) outcomes for people with dementia and carers (the delivery tier of the programme theory), individual case studies will be conducted. Persons with dementia and carer dyads recruited together will serve as the case study unit for analysis. If the person with dementia is recruited on their own, they will form the unit for analysis on their own (maximum of 90 case studies recruited, with the expectation numbers will decrease owing to attrition caused by death, participants moving out of the area, and people with dementia moving into care homes permanently). Please see Box 2 for an example of a programme theory statement from the delivery tier.

Box 2 **Definition of the CCMO heuristic using a delivery tier statement example. CCMO = context – (intervention) component – mechanism – outcome. DSW = dementia support worker.**

**Context** refers to existing personal, structural, and organisational factors that may influence the ways in which an intervention is delivered and responded to by different individuals.

*Example:* Person with dementia and/or carer has comorbid conditions and/or a complex medical history before meeting with DSW. To date, their care has been uncoordinated and they are unsure of how to navigate the health and social care system.

**Components** are specific actions within an intervention that are delivered to participants.

*Example:* IF the DSW supports the person with dementia and carer to engage with their GP surgery/other health and social care professionals/the voluntary sector within their community.

**Mechanisms** are the underlying causal processes, the ways in which individuals reason in response to intervention components, that must be ‘activated’ or ‘triggered’ in conducive contexts in order for ‘outcomes’ to be attained.

*Example:* THEN the person with dementia and/or carer will start to feel more positive about navigating the system/future interactions with health and care professionals (including transitions in care).

**Proximal outcomes** are the observable changes to intervention recipient’s behaviour.

*Examples:*

THEN the person with dementia and/or carer will enact new coping strategies related to seeking and engaging with a wider care team.

THEN the person with dementia and/or carer will disclose more to other professionals involved in their care.

**Distal outcomes** are medium to longer-term outcomes that can be behavioural but can also relate to changes in physical health and wellbeing for an individual and service-based changes (for example, in terms of the workforce, quality of care, efficiency, and coordination).

##### Coding framework

The coding process utilises a realist logic, which ensures generative causation can be examined. The entire EPT (the delivery and facilitation tier of the programme theory; please see Supplementary Box S2 to review programme theory) will form the framework for coding of case study data, which will be managed in NVivo (version 12). The EPT consists of a collection of realist statements (organised under core domains of the programme theory; for example, engagement and disclosure, collaboration, and peer support) that have been constructed using an expanded variation of the traditional ‘CMO’ heuristic: context (C) – (intervention) component (C) – mechanism (M) – outcome (O). An expanded heuristic was used as existing realist evaluations have shown that they can provide a deeper analysis into the individual components of a complex intervention,^19^ providing clarity on what casual explanation can be attributed to specific intervention actions (or strategies, components, or resources).^20^ Another reason for using this (expanded) CCMO within the evaluation was to aid consistency in coding, analysis, and dissemination by clearly defining what is meant by the term ‘mechanism’ and 'outcome'.

To date, there have been varying (or missing) definitions of ‘mechanism’ used within published realist evaluations.^20–22^ Based on coding experiences in phase 1, it was determined that it would be easier for researchers to code data to the EPT, if the two aspects of a (traditionally defined) ‘mechanism’ (resource and response) were split into two separate elements, as others^22^ have also advocated (see Box 2).

In addition, the authors wanted to distinguish between responses to intervention components (resources) that involve 1) a generative change in the targeted person’s internal reasoning (for example, changes in beliefs or thoughts, emotions, and understanding), which can alter their decision making,^23^ as a type of mechanism; and 2) responsive changes in behaviour or actions. Some realist evaluations have viewed both types of responses as mechanisms, whereas others have only focused on one type of response (or chosen to define mechanisms differently);^24^ it was chosen to only use the former type of response (reasoning) to define mechanisms, as the latter was for D-PACT purposes a (proximal) outcome of the intervention, and it enabled how changes in reasoning led to changes in behaviour to be examined more clearly.

In addition to theorising about proximal outcomes, it was hypothesised what medium and longer-term (distal) outcomes the intervention would result in. Please see Box 2 for the definition of context, component, mechanism, and outcomes (proximal and distal) within the evaluation, along with an example of a CCMO statement from the current programme. Evidencing a realist theory of the distal and proximal outcomes will enable the authors to develop a comprehensive evidence base and framework that will help define more fully the outcomes for the different actors, in different settings, and across organisational levels and sectors; that is, the shift in focus from outcomes to value and impact (aim 2). By evidencing proximal, as well as distal outcomes, the study will provide further insight into how those long-term and far-reaching changes occur.

##### Coding process

Data pertaining to delivery and/or mechanism or outcome attainment will be coded to the relevant part of each CCMO statement. The framework and coding process has been designed to enable both deductive coding (capturing qualitative data that provide insights related to each statement within the existing EPT) and inductive coding (capturing new insights, meaning, or refinements to existing theory).

The following coding process will be used. Researchers will:

- Initially identify instances of an intervention component within the data.
- Examine whether evidence of influencing context, mechanism triggering, and/or (proximal) outcome attainment can be observed in the data in response to the delivery of that specific (intervention) component, and, if so, subsequently code that data deductively or inductively to the coding framework.
- Explore whether data should be coded to existing ‘unintended’ programme theory statements (developed through phase 1). Statements labelled ‘unintended’ depict instances when the delivery of an (intervention) component, with a certain context, triggers an unnintended mechanism and, as a result, leads to an unintended outcome. Inductive data coded to individual intervention components may result in further theory statements capturing unintended mechanisms and outcomes being developed in the analysis phase.
- Assess whether the data source contains relevant information that should be added to an overall context summary memo for that specific case.
- Code data linking context, mechanism(s), and outcome(s) all together to both the mechanism(s) and outcome(s) child nodes for the specific intervention component they relate to. This will enable the analysis to utilise data that shows evidence relating to both full and incomplete CCMOs — with incomplete CCMOs being potentially ‘completed’ by other data sources collected at that time or from a cumulative picture of what mechanisms and outcomes were reported under each intervention domain/strategy over time.
- In addition to coding proximal outcomes, code relevant data (related to medium–longer-term outcomes) to a child node labelled ‘distal outcomes’ — placed under the parent node denoting the intervention component that led to that change. Such insights may be more observable in data collected toward the end of the evaluation but may also be present in earlier data.

Researchers are receiving ongoing training about how to code and a coding manual (see Supplementary Information S1 for the delivery tier version of the codebook). Researchers will meet on a regular basis to review, discuss, and compare their coding, resolving any inconsistencies in how they are coding and/or misunderstandings about the process.

##### Within-case analyses

Once all the data have been coded for an individual case study, a within-case analysis will be undertaken. This will be done within two matrix templates (one to organise each person with dementia and carer’s coded data to the CCMO statements for the delivery tier of the EPT, and one to organise practitioner’s coded data to the CCMO statements within the facilitation EPT) developed within Microsoft Excel or Word. When a carer and person with dementia are recruited together their data will be entered into a combined, dyadic matrix, which has a split carer and person with dementia column for each CCMO so that differences or similarities between CCMO occurrences for both members of the dyad can be observed.

The matrices will enable researchers to create a trajectory of change for each case by exploring, through vertical columns, how individual CCMO occurrences may have reoccurred or changed over time and whether individual CCMOs may have been more likely to occur at certain time points; that is, early or later in the intervention. It will also allow the researcher to pull together data sources from similar time points to get a better understanding of the interaction within and between individual CCMOs (for example, ripple effects).^25^ Some data sources may provide more insight in one aspect of the CCMO configuration, and the combining of evidence from different data sources will reinforce or challenge the evidence the data sources provide individually.

Researchers will review DSW timesheets, intervention tools, and participant medical records for the time periods where a CCMO was evidenced to determine whether evidence from these data sources could be used to corroborate or challenge the existing qualitative evidence within the matrix. When data from these additional sources appear to link to CCMOs within the matrix, that data will be added to the matrix. A small proportion of CCMO statements in the facilitation tier relate to other actors and recipients; for example, GP surgeries and DSW supervisors. Data coded to these statements will be amalgamated and analysed separately to explore context-dependent mechanism activation, and any potential patterns in barriers or facilitators, in order to produce qualitatively generalisable insights.

Responses to items on the participants’ outcome and experience measures, collected at three different time points (T0 [pre-intervention; baseline], T1 [4–6 months of intervention support], and T2 [9–12 months of intervention support]), will be added to each participant’s (people with dementia and carer) delivery matrix. This will allow the authors to further triangulate the data and consider links between evidenced occurrences of CCMOs up to the time of measurement data being collected (at each of the three time points) and the findings from the measurement scales. Summaries of what CCMOs were evidenced or not within the case matrices will be created for later use (see finalisation of the programme theory).

##### Cross-case analyses

Researchers will review whether to conduct cross-case analyses by either using a matrix method — utilising the existing ‘within-case’ matrices created through the within-case analyses detailed above — or through the creation of one master NVivo file. The master NVivo file would contain each participant’s coded data and would enable the use of matrix coding queries and node matrices to examine how coding for individual CCMO statements were distributed across the entire dataset and for certain groupings of case studies; for example, case studies from the same site, case studies from the same type of participant (carer or person with dementia, or DSW), with the same ethnic group, age group, socioeconomic background, or professional background (for DSW), and so on. In addition, participant groups will be able to be formed for cross-case analysis by: 1) using interpreted ‘themes’ occurring throughout individuals’ overall case context memos (see coding process section for more detail); and 2) by using total and individual question scores from completed measures (for example, participants who all scored low or high on feeling like a burden, or participants who scored highly on experience of care). ‘Pilot’ cross-case analyses, the number of people recruited onto the study, and the amount of time left for cross-case analyses will inform the decision on which method to use.

Through this process the researchers will identify the following:

- who experienced certain mechanisms and outcomes, triggered by intervention components, within a particular context;
- in what contexts the intervention components were not delivered or did not trigger intended mechanisms or led to beneficial outcomes; and
- whether people who reported medium- and longer-term changes (positive or negative to their wellbeing, experiences of care, independence, and engagement) experienced certain CCMOs more often than others and whether there were similar contextual factors shared by these subgroups (for example, their ethnic background, type of dementia, age, and type of community).

Researchers will then return to the within-case matrices and summaries, using retroductive analyses^26^ to explore possible reasons for differences found between different contexts. The aim is to understand general patterns across cases on how outcomes can be obtained for people from different contextual backgrounds. If there are variations in recruitment progress across sites, resulting in varied amounts of data collected and coded at the time of cross site analysis, only partial cross-case analyses for some aspects of the programme theory may be feasible.

### 2. Conversation analysis of video recordings of D-PACT intervention sessions from a subsample of case studies

Conversation analysis (CA) can investigate how professionals and patients or clients communicate during certain activities.^27^ While it does not attend to informants’ internal cognitive or emotional states, it does micro-analyse, using recordings of real-time interactions, what people say and how they say it, and how this enables social actions to be achieved through communication (for example, how a request is made). CA focuses on how participants negotiate shared understanding, and how all members of an interaction (for example, patient or client and professional) shape the trajectory of the interaction, on a turn (of speakership) by turn basis. As such, CA, offers an opportunity to examine how specific interactional (intervention) components of interest are enacted in intervention sessions, for example, how co-setting an agenda at the start of a support meeting may impact on patient participation, for example, a person with dementia topicalising issues for discussion.

Within the evaluation, CA is being applied to develop a more in-depth micro-level programme theory, specific to the interactional components and intended proximal outcomes (interaction behaviours of the intervention recipients) of the intervention to enable a better ‘practice-level’ understanding of how the intervention works. Video recordings of support sessions from a subset of people with dementia and carer-case studies will be used to refine interactional aspects of the delivery EPT, and a collection of supervision recordings will be used to refine the interactional aspects of the facilitation EPT.

By analysing other data sources (for example, realist interviews, diary data, and brief interviews straight after a recorded support meeting), the researcher may additionally be able to link communication practices to what internal responses participants experienced; this possibility will be explored during the evaluation.

### 3. An assessment of quantitative outcome and experience measures over time

Quantitative data are being collected at three time points: T0 (pre-intervention), T1 (at 4–6 months), and T2 (at 9–12 months). These data will explore changes over time for people with dementia and their carers using outcome and experience measures (see Supplementary Box S1), which were tested in terms of their suitability and feasibility in phase 1.

Baseline characteristics will be summarised using mean (standard deviation [SD]) or median (interquartile range [IQR]) for continuous variables, and *n* (%) for binary or categorical variables. For the outcome measures, summary statistics will be presented using mean (SD) or medians (IQR) at each time point, and for the change in scores from baseline, with corresponding 95% confidence intervals. Graphs tracking the trajectory of participants will be used to visualise changes in outcome and experience measures over time. If there are sufficient data, from a large enough sample, exploratory models for repeated measures data will be fitted and these will explore within-subject (time-point) and between-subject effects (key baseline covariates) on the outcome. If there are not sufficient data for models, it will be tested whether outcome and experience measures differ according to variables of interest using simpler correlations and tests of significant differences.

### 4. Exploratory health economics analysis

There will be two parts to the health economics (HE) analysis. The first will employ a traditional HE approach, while the second will be exploratory, using a realist approach.

#### a) Cost of delivery and associated resource utilisation

The estimation of direct costs associated with delivering the D-PACT intervention for the whole system and for a range of individuals will be carried out in the following two ways:

- at a whole-team level: estimating costs through the employed time of DSW and supervisor (not including time on research tasks) based on their NHS agenda for change grades; and
- at individual-patient level: through analysing the DSW timesheets to apportion above costs to individuals and to specific D-PACT tasks.

Additionally, wider costs of health and social care, and carer costs will be described and estimated from both the resource use questionnaires administered to people with dementia and carers, and data from practice electronic health records.

#### b) Assessment of contribution of D-PACT to changing the trajectory of high-cost events or near misses

A realist cognisant, mixed-methods economic analysis will be conducted to examine the relationship between the intervention delivery and relatively rare but high-cost health and social care utilisation events or ‘near misses’ — avoidance of a negative event or unwanted change in care resulting from a sudden escalation in the patient’s needs or risk (acute admissions, safeguarding, and nursing home care). The rationale for developing this realist economic (RE) analysis is that high-cost events are relatively rare and should be analysed in isolation as they can distort economic results; a detailed qualitative realist analysis of care might provide evidence as to whether D-PACT activity is directed to changing the trajectory of such events, or whether the care actually contributed.

Admissions to hospital, nursing, or care home (or near misses) will be identified from patients’ (only people living with participants with dementia) electronic medical records (held at GP surgeries), using a search and extraction protocol developed from feasibility work using a small sample (four patient records from a GP surgery at each site), but which can be refined if necessary. These records, DSW case notes (made during the 12 months of delivering the intervention for each participant), and qualitative data (coded as part of the case study analyses) will then be examined to explore evidence for impact of the anticipatory care component of D-PACT (for example, the DSW developing a care plan that was then used and the DSW engaging GPs in active acute care). This review of records will be supplemented with qualitative follow-up interviews with practitioners and/or carers if required. This RE analysis will incorporate analytic strategies described in the longitudinal case study analysis above.

### Synthesis

A mixed-methods approach will be used to examine the findings from each strand of evaluation analyses to expand the programme theory and evaluate the intervention’s value and impact (aim 2). Bringing together analytic streams through the use of a bespoke co-developed joint display table^18^ focused on causal effects that generate value and impact at micro-, meso-, and macro-levels will enhance stakeholders’ ability to make judgements about the likely benefit of the intervention.

Findings from each analysis stream will be summarised in joint display tables, with categories based on both pre-determined aspects of value and impact (for example, reach; existing, evidenced programme theory statements regarding proximal and distal outcomes; and inductively developed aspects of value and impact [developed through this analysis and stakeholder engagement]). The tables will also be organised according to the following: 1) facilitation and delivery categories, to identify processes affecting DSWs and study participants; 2) proximal and distal outcomes; and 3) micro-, meso-, and macro-levels. Throughout this process, patterns within the joint display table, including areas of agreement or discordance, will be examined through triangulation of the data (underpinning each analysis strand’s findings) through a convergent mixed-methods model.^28^

This additional layer of analysis will involve stakeholder and colleagues’ input into the design of the joint display table and the definition of value and impact that is used (for example, their relatability to the real world) and their interrogation of the process (for example, has a clear analytic process been followed? Could another explanation for how value and impact was achieved be given?). Colleagues will include members of the wider team who are less closely tied to the data but have an understanding of the programme theory; stakeholders will include professionals and the patient and public involvement group. The analysis will also involve the use of a bespoke self-appraisal tool, informed by realist^29^ mixed-method approaches^30^ to assessing the transparency, rigour, and quality of both the evidence and the analytic process used.

## Discussion

The proposed evaluation will fill gaps in current evidence on how post-diagnostic support based in primary care for people with dementia and their carers works, who such support works for, and in what circumstances. These data will support decisions around post-diagnostic support for people with dementia and their carers. The programme theory not only examines the delivery of such support, but also the facilitation of those who provide that support. This will have implications for workforce training and support.

The study will also demonstrate how to apply a realist design to evaluations where a randomised controlled trial (RCT) design is less appropriate, in line with latest MRC guidance on the evaluation of complex behavioural interventions and their impact.^13^ For example, it is increasingly being questioned whether the RCT, or an RCT alone, is the most appropriate method for evaluating complex healthcare interventions owing to their lack of sensitivity to varying contexts, the length and complexity of the causal chains linking the intervention with outcomes,^31,32^ problems with recruitment owing to unengaging trial procedures, and concerns as to whether standard outcome measures will be sensitive to the varied and unpredictable achievements generated by person-centred interventions.^16,33^ The longitudinal mixed-methods (non-randomised) realist evaluation design is sufficiently flexible to support adjustable recruitment processes. Experiences and data from recruitment in phase 1 showed that flexibility was necessary for person-centred recruitment that went at the pace of the person with dementia and could be adjusted to suit other needs. This design provides the same flexibility when collecting responses to the outcome and experience (questionnaire) measures, where even those who are in more advanced stages of dementia can be supported to respond (thus preserving their voice as much as possible). An RCT design would have necessitated adhering to rigid processes for how people living with dementia could respond to binary questioning or the use of a full proxy. This design also more easily accommodates a person-centred, highly flexible intervention like D-PACT, which is constructed to be responsive and adaptable to individual needs; for example, bringing them into care during increased times of perceived need. In this way, real-world delivery of complex health and social care services is mirrored, which facilitates an examination of the wider system in which this care would sit. Participants benefit too from adaptable research processes and within-evaluation practitioner feedback sessions (that involve discussion of emerging findings). Such sessions will enable timely and necessary changes to be made to intervention delivery, ensuring the intervention responds to, and caters for, different and evolving situations and needs. The novel approach places greater importance of qualitative data and commits to integrating high-volume and longitudinal qualitative data *with* quantitative data during analyses to generate a breadth of evidence about what impact and value the intervention can potentially have (and *how*) at various levels of care. This contributes to the growing openness to alternative approaches (including realist ones) to evaluating complex interventions.^13,31^ The work also addresses the question of whether such approaches to evaluation do enough to establish the value and impact of an intervention.

It is acknowledged that the lack of a randomised control group reduces the ability to definitively compare outcomes with and without support; however, it is felt the advantages of using a realist evaluation design outweigh those of using an RCT for this particular project.

## References

[1] Dowrick A, Southern A (2014). Dementia 2014: opportunity for change. https://www.alzheimers.org.uk/sites/default/files/migrate/downloads/dementia_2014_opportunity_for_change.pdf.

[2] HM Government (2022). Health and Care Act 2022. https://www.legislation.gov.uk/ukpga/2022/31/contents/enacted.

[3] Hossain MZ (2021). The costs of caregiving: exploring the perceived burden of dementia among Bangladeshi caregivers in Britain. Am J Fam Ther.

[4] Peel E, Harding R (2014). 'It's a huge maze, the system, it's a terrible maze': dementia Carers' constructions of navigating health and social care services. Dementia (London).

[5] Iliffe S, Kendrick D, Morris R, Masud T (2014). Multicentre cluster randomised trial comparing a community group exercise programme and home-based exercise with usual care for people aged 65 years and over in primary care. Health Technol Assess.

[6] Karlsson S, Bleijlevens M, Roe B (2015). Dementia care in European countries, from the perspective of people with dementia and their caregivers. J Adv Nurs.

[7] Alzheimer’s Society (2016). Dementia advisors: a cost-effective approach to delivering integrated dementia care. https://www.alzheimers.org.uk/sites/default/files/migrate/downloads/dementia_advisers_a_cost_effective_approach_to_delivering_integrated_dementia_care.pdf.

[8] Department of Health Policy Paper (2016). Joint declaration on post-diagnostic dementia care and support. https://www.gov.uk/government/publications/dementia-post-diagnostic-care-and-support/dementia-post-diagnostic-care-and-support.

[9] Pawson R, Tilley N (1997). Realistic Evaluation.

[10] Richards DA, Bazeley P, Borglin G (2019). Integrating quantitative and qualitative data and findings when undertaking randomised controlled trials. BMJ Open.

[11] Westhorp G (2014). Realist impact evaluation: an introduction. https://cdn.odi.org/media/documents/9138.pdf.

[12] Wong G, Greenhalgh T, Westhorp G (2013). RAMESES publication standards: realist syntheses. BMC Med.

[13] Skivington K, Matthews L, Simpson SA, Craig P (2021). A new framework for developing and evaluating complex interventions: update of medical research Council guidance. BMJ.

[14] Moore GF, Audrey S, Barker M (2015). Process evaluation of complex interventions: Medical Research Council guidance. BMJ.

[15] Griffiths S, Weston L, Morgan-Trimmer S (2022). Engaging Stakeholders in realist programme theory building: insights from the prospective phase of a primary care dementia support study. Int J Qual Methods.

[16] Weston L, Rybczynska-Bunt S, Quinn C (2022). Interrogating intervention delivery and participants' emotional states to improve engagement and implementation: a realist informed multiple case study evaluation of Engager. PLoS One.

[17] Oh TM, Batool S (In press.). Recruitment of people with dementia and their Carers to a complex intervention study in a pandemic: learning from the dementia Personalised care team (D-PACT) feasibility study.

[18] Guetterman TC, Fetters MD, Creswell JW (2015). Integrating quantitative and qualitative results in health science mixed methods research through joint displays. Ann Fam Med.

[19] Abejirinde I-O, Zweekhorst M, Bardají A (2018). Unveiling the black box of diagnostic and clinical decision support systems for Antenatal care: realist evaluation. JMIR Mhealth Uhealth.

[20] De Weger E, Van Vooren NJE, Wong G (2020). What’s in a realist configuration? Deciding which causal configurations to use, how, and why. Int J Qual Methods.

[21] Marchal B, van Belle S, van Olmen J (2012). Is realist evaluation keeping its promise? A review of published empirical studies in the field of health systems research. Evaluation.

[22] Dalkin SM, Greenhalgh J, Jones D (2015). What's in a mechanism? Development of a key concept in realist evaluation. Implement Sci.

[23] Mukumbang FC, Van Belle S, Marchal B, van Wyk B (2017). Exploring ‘generative mechanisms’ of the antiretroviral adherence club intervention using the realist approach: a scoping review of research-based antiretroviral treatment adherence theories. BMC Public Health.

[24] Lemire S, Kwako A, Nielsen SB (2020). What is this thing called a mechanism? Findings from a review of realist evaluations. New Dir Eval.

[25] Jagosh J, Bush PL, Salsberg J (2015). A realist evaluation of community-based participatory research: partnership synergy, trust building and related ripple effects. BMC Public Health.

[26] Mukumbang FC, Kabongo EM, Eastwood JG (2021). Examining the application of Retroductive theorizing in realist-informed studies. Int J Qual Methods.

[27] Barnes RK (2019). Conversation analysis of communication in medical care: description and beyond. Res Lang Soc Interact.

[28] Turner SF, Cardinal LB, Burton RM (2017). Research design for mixed methods: a triangulation-based framework and roadmap. Organ Res Methods.

[29] Rybczynska-Bunt S, Weston L, Byng R (2021). Clarifying realist analytic and interdisciplinary consensus processes in a complex health intervention: a worked example of judgemental rationality in action. Evaluation.

[30] O’Cathain A, Tashakkori A, Teddlie C (2010). SAGE Handbook of Mixed Methods in Social and Behavioral Research.

[31] Bonell CP, Hargreaves J, Cousens S (2011). Alternatives to randomisation in the evaluation of public health interventions: design challenges and solutions. J Epidemiol Community Health.

[32] Deaton A, Cartwright N (2018). Understanding and misunderstanding randomized controlled trials. Soc Sci Med.

[33] Byng R, Lennox C, Kirkpatrick T (2022). Development and evaluation of a collaborative care intervention for male prison leavers with mental health problems: the Engager research programme.

